# Listeriosis Outbreaks and Associated Food Vehicles, United States, 1998–2008

**DOI:** 10.3201/eid1901.120393

**Published:** 2013-01

**Authors:** Emily J. Cartwright, Kelly A. Jackson, Shacara D. Johnson, Lewis M. Graves, Benjamin J. Silk, Barbara E. Mahon

**Affiliations:** Author affiliation: Centers for Disease Control and Prevention, Atlanta, Georgia, USA

**Keywords:** foodborne illness, food vehicles, outbreaks, listeriosis, bacteria, Listeria monocytogenes, United States

## Abstract

*Listeria monocytogenes*, a bacterial foodborne pathogen, can cause meningitis, bacteremia, and complications during pregnancy. This report summarizes listeriosis outbreaks reported to the Foodborne Disease Outbreak Surveillance System of the Centers for Disease Control and Prevention during 1998–2008. The study period includes the advent of PulseNet (a national molecular subtyping network for outbreak detection) in 1998 and the *Listeria* Initiative (enhanced surveillance for outbreak investigation) in 2004. Twenty-four confirmed listeriosis outbreaks were reported during 1998–2008, resulting in 359 illnesses, 215 hospitalizations, and 38 deaths. Outbreaks earlier in the study period were generally larger and longer. Serotype 4b caused the largest number of outbreaks and outbreak-associated cases. Ready-to-eat meats caused more early outbreaks, and novel vehicles (i.e., sprouts, taco/nacho salad) were associated with outbreaks later in the study period. These changes may reflect the effect of PulseNet and the *Listeria* Initiative and regulatory initiatives designed to prevent contamination in ready-to-eat meat and poultry products.

## Abstract 2

Outbreak investigations can identify industrial gaps and regulatory measures to protect food.

*Listeria monocytogenes* is transmitted predominantly through contaminated food (*1*). Listeriosis includes a spectrum of clinical illnesses ranging from febrile gastroenteritis to potentially fatal bacteremia and meningitis in groups at higher risk for invasive disease, including older adults and persons with certain medical conditions (*2*,*3*). Although pregnant women infected with *L. monocytogenes* typically experience a mild influenza-like illness or an asymptomatic infection, pregnancy-associated listeriosis can result in fetal loss, preterm delivery, invasive neonatal infection, and infant death.

The Centers for Disease Control and Prevention (CDC) estimates that 1,662 invasive infections with *L. monocytogenes* occur annually in the United States, causing 1,520 hospitalizations and 266 related deaths (*4*). Population-based surveillance demonstrated a 24% decrease in the crude incidence of laboratory-confirmed listeriosis from 0.41 to 0.31 cases per 100,000 population during 1996–2003 (*5*). Since 2003, the incidence of listeriosis has remained stable, with rates ranging from 0.25 to 0.32 cases per 100,000 population during 2004–2009. The 6-year average rates of hospitalization and death were 0.26 hospital admissions and 0.05 deaths or fetal losses per 100,000, respectively (*6*). Millions of US dollars in health care expenditures and quality-adjusted life years are lost to invasive listeriosis annually (*7*).

Foodborne transmission of listeriosis was first recognized conclusively after an outbreak in Canada in 1981 that was associated with consumption of contaminated coleslaw (*1*). In the United States, the first recognized foodborne listeriosis outbreak occurred in 1983 and was associated with pasteurized milk (*8*). During 1983–1998, outbreaks of foodborne listeriosis associated with Mexican-style cheese (*9*) and shrimp (*10*) were subsequently documented; a single case was also attributed to turkey frankfurters (*11*).

PulseNet, the national molecular subtyping network for enteric bacterial disease surveillance, was established in 1998 (www.cdc.gov/pulsenet). *L. monocytogenes* isolates from patients are sent to state public health laboratories for standardized pulsed-field gel electrophoresis (PFGE); the PFGE patterns are then uploaded to a central database (PulseNet) for national comparisons (*12*). When >2 *L. monocytogenes* isolates with indistinguishable PFGE pattern combinations are uploaded within a 120-day period, this cluster is evaluated. An investigation is initiated if the upload rate for this pattern combination is greater than the historical background or if other epidemiologic indicators suggest a common source.

Invasive listeriosis has been a nationally notifiable disease in the United States since 2001. Although most listeriosis cases are sporadic (i.e., not associated with a recognized cluster of illness), the detection of a listeriosis outbreak is a critical opportunity to prevent additional illness and death by removing a contaminated vehicle from the food supply. In addition, outbreak investigations often provide information about transmission of *L. monocytogenes* that can be used to improve food safety (*13*). However, epidemiologic investigations of listeriosis clusters are challenging because they are typically detected as a small number of geographically dispersed case-patients (some of whom may have died), and the incubation period can be lengthy, making patients’ recall of food exposures difficult (*14*).

Following a 2003 Council of State and Territorial Epidemiologists position statement, the *Listeria* Initiative was launched in 2004 to address these concerns (www.cdc.gov/listeria/surveillance.html). The *Listeria* Initiative encourages state and local health department officials to routinely interview all patients with culture-confirmed listeriosis as soon as they are reported by using a standardized, extended questionnaire to collect food histories. Concurrently, clinical isolates are submitted to public health laboratories for PFGE subtyping and submission to PulseNet, and PFGE results are linked to epidemiologic information in the *Listeria* Initiative database. When a cluster is identified in PulseNet, *Listeria* Initiative data related to that cluster can be reviewed quickly to identify common food exposures. The *Listeria* Initiative also facilitates case–case studies by comparing exposures reported by cluster-associated cases with information from listeriosis cases that are not associated with the cluster. The effectiveness of the case–case approach has been illustrated repeatedly, for example, during the investigation of large, multistate outbreaks associated with delicatessen turkey meat and cantaloupe (*15*,*16*).

This report summarizes single-state and multistate listeriosis outbreaks reported to CDC during 1998–2008. We describe characteristics of the outbreaks and affected patients to summarize outbreak trends, *L. monocytogenes* serotype distribution, and implicated foods.

## Outbreak Identification and Characterization

To identify all listeriosis outbreaks reported during 1998–2008 in the United States, we reviewed data from the CDC Foodborne Disease Outbreak Surveillance System (FDOSS). FDOSS is a national surveillance system through which state, local, tribal, and territorial health departments voluntarily submit to CDC reports of outbreaks by using a standardized form (CDC form 52.13) (*17*). In 1998, FDOSS surveillance activities were enhanced through the use of an electronic data collection form and other activities to increase reporting. For each outbreak, FDOSS captures information on etiology, food vehicle, outbreak size, duration, geographic location, setting, and selected outcomes (i.e., number of illnesses, hospitalizations, and deaths). Aggregated age group and sex data are also reported. A listeriosis outbreak was defined as >2 listeriosis cases linked to a common source by a public health investigation. A listeriosis outbreak was considered confirmed if the same serotype of *L. monocytogenes* was isolated from >2 patients exposed to either epidemiologically implicated food or food from which the same serotype was isolated. Outbreaks were considered to be multistate if exposure to the implicated food occurred in >1 state.

Hospitalization (number of hospitalized cases/total cases) and case-fatality rates (CFR) (number of deaths/total cases) were calculated for the study period. When >50% of demographic data were missing, remaining data were not analyzed. Outbreak duration was calculated as the number of days between the dates of illness onset of the first and the last reported cases. To define early (1998–2003) and late (2004–2008) study periods, 2004 was selected as a cutoff point because it coincides with the launch of the *Listeria* Initiative. Serotyping of outbreak-associated *L. monocytogenes* isolates was performed by CDC (*18*). Serotype information was matched to each outbreak reported in FDOSS. In addition, we conducted a systematic literature review of published reports of listeriosis outbreaks by using 5 electronic databases (PubMed, Embase, Web of Science, Toxnet, and CAB Direct) and medical subject headings “*Listeria monocytogenes*,” “*Listeria* infections,” “listeriosis,” “disease outbreaks,” and “foodborne diseases.” When discrepancies were identified between published reports and FDOSS, the published data were used.

## Outbreaks and Associated Food Vehicles

**Figure 1:**
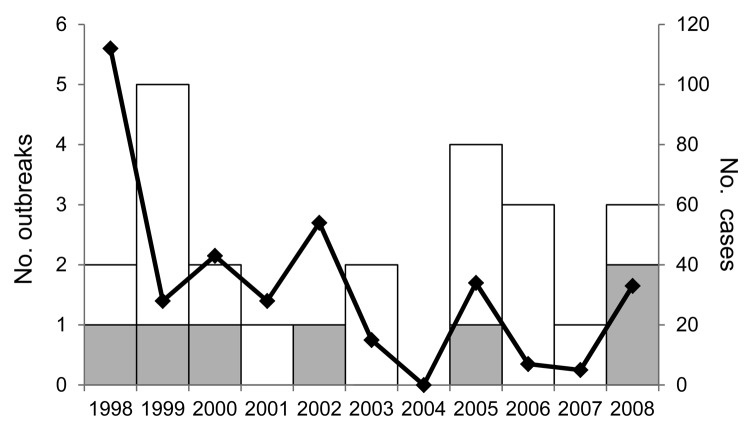
Reported listeriosis outbreaks by single-state or multistate status and total number of outbreak-associated cases, Foodborne Disease Outbreak Surveillance System, United States, 1998–2008 (n = 24 outbreaks). White bar sections indicate single state-and multistate outbreaks, gray bar sections indicate multistate outbreaks, and black line indicates total ill.

**Figure 2:**
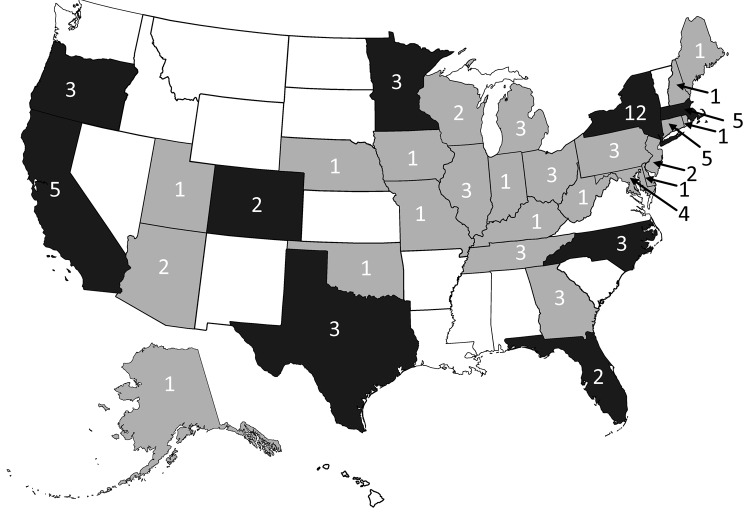
Distribution of single-state single and multistate outbreaks of listeriosis, 1998–2008, Foodborne Disease Outbreak Surveillance System, United States, 1998–2008 (n = 24 outbreaks). Dark gray indicates single-state and multistate outbreaks, and light gray indicates multistate outbreaks. Values indicate total outbreaks in each state. The grand total of outbreaks indicated in each state is greater than 24 because of multistate outbreaks.

During 1998–2008, a total of 26 listeriosis outbreaks were reported to FDOSS; 24 were confirmed (Figure 1; Table 1). Eight (33%) of these outbreaks were described in the published literature (*14*,*15*,*19*–*24*). The 24 confirmed outbreaks resulted in 359 illnesses, 215 hospitalizations, and 38 deaths. The 11-year hospitalization rate was 60%, and the CFR was 11% (Table 2). Among 16 outbreaks with available data, the median duration was 42 days (range 1–389 days). Seven (29%) multistate outbreaks were reported, and 33 states reported cases in multistate outbreaks (Figure 2). Nine states reported single-state outbreaks. New York reported more listeriosis outbreaks than any other state (n = 6).

**Table 1:** Reported listeriosis outbreaks (n = 24) by year, Foodborne Disease Outbreak Surveillance System, United States, 1998–2008*

Study period, year†	Multistate	Duration, d	Total no. cases‡	No. hospitalizations	No. deaths	*Listeria monocytogenes* serotype	Food vehicle (reference)
Early							
1998	Yes	389	108	101	14	4b	Frankfurters (*19*)
	No	NA	4	NA	NA	NA	Frankfurters
1999	No	NA	6	NA	NA	NA	Unknown
	No	NA	4	NA	NA	1/2a	Frankfurters
	No	NA	5	5	1	NA	Deli meat
	Yes	NA	11	NA	NA	1/2a	Pâté
	No	NA	2	2	1	NA	Deli meat
2000	No	122	13	13	0	4b	Mexican-style cheese (*22*)
	Yes	151	30	29	4	1/2a	Deli meat (*14*)
2001	No	3	28	0	0	1/2a	Deli meat (*21*)
2002	Yes	100	54	NA	8	4b	Deli meat (*15*)
2003	No	NA	3	NA	NA	4b	Unknown
	No	NA	12	12	1	4b	Mexican-style cheese
Late							
2005	No	32	6	6	0	4b	Unknown
	No	7	3	3	0	1/2b	Grilled chicken
	Yes	37	13	13	1	1/2a	Deli meat
	No	36	12	12	0	4b	Mexican-style cheese
2006	No	7	2	1	1	4b	Unknown
	No	2	2	0	0	1/2b	Taco or nacho salad
	No	1	3	2	1	4b	Cheese
2007	No	163	5	5	3	4b	Milk (*20*)
2008	No	47	5	5	3	1/2a	Tuna salad (*23*)
	Yes	351	20	2	0	1/2a	Sprouts
	Yes	150	8	4	0	1/2a	Mexican-style cheese (*24*)

*NA, no data available. †No listeriosis cases were reported in 2004. ‡Includes laboratory-confirmed and epidemiologically linked cases.

**Table 2:** Characteristics of 24 listeriosis outbreaks by implicated food categories, Foodborne Disease Outbreak Surveillance System, United States, 1998–2008*

Food category	No. outbreaks	Total no. cases†	No. (%) hospitalized	No. deaths (CFR, %)	Age group, y, no. (%)‡			No. (%) female‡
					<1	1–49	>50	
Deli meats	6	132	49 (37)	15 (11)	NA	NA	NA	40 (45)
Frankfurters	3	116	101 (87)	14 (12)	0	52 (46)	60 (54)	57 (49)
Other meats	2	14	3 (21)	0	NA	NA	NA	2 (67)
Mexican-style cheese	4	45	41 (91)	1 (2)	8 (18)	32 (73)	4 (9)	37 (84)
Other dairy products	2	8	7 (88)	4 (50)	1 (13)	1 (13)	6 (75)	4 (50)
Salad/other	3	27	7 (26)	3 (11)	0	7 (26)	20 (74)	16 (59)
Unknown	4	17	7 (41)	1 (6)	0	1 (13)	7 (88)	4 (50)
Overall	24	359	215 (60)	38 (11)	10 (4)	120 (50)	108 (45)	160 (54)

*CFR, case-fatality rate; NA, not available. †Includes laboratory-confirmed and epidemiologically linked cases. ‡Percentages were calculated on the basis of available information on age distribution (17 outbreaks) and sex (19 outbreaks) for 238 and 296 patients, respectively.

 Information on the age distribution of ill persons was available for 17 (71%) of the 24 outbreaks. These 17 outbreaks included 238 patients (66% of all illnesses associated with the 24 outbreaks), including 10 (4%) infants, 120 (50%) persons 1–49 years of age, and 108 (45%) >50 years of age. Information on sex was available for 19 (79%) of the 24 outbreaks. Among 296 patients with sex reported, 160 (54%) were female (Table 2).

Although most of the outbreaks occurred in community settings (i.e., foods consumed in private homes, commercial establishments, or both), 2 outbreaks were attributed to foods prepared and served in hospitals. A food vehicle was implicated in 20 (83%) of the 24 confirmed listeriosis outbreaks (Table 2). Six (25%) outbreaks were attributed to delicatessen (deli) meats. Three (13%) outbreaks, including the largest outbreak (n = 108 cases) during the study period, were associated with frankfurters. Mexican-style cheese (queso fresco or queso blanco) was implicated in 4 (17%) outbreaks. Three outbreaks involved Mexican-style cheese made from unpasteurized milk, and 1 outbreak was associated with cheese made from pasteurized milk (*24*). Outbreaks caused by Mexican-style cheese primarily affected women (84%); most (73%) patients were 1–49 years of age.

Two outbreaks involved other dairy products, including cheese made from pasteurized sheep’s milk and flavored, pasteurized milk. Environmental investigations conducted during these 2 dairy-associated outbreaks (*20*,*24*) found that milk had been adequately pasteurized. Thus, postpasteurization contamination was believed to be the contributing factor for both outbreaks. Other implicated food vehicles included taco/nacho salad, tuna salad, and sprouts.

*L. monocytogenes* serotype information was available for 20 (83%) of the 24 confirmed listeriosis outbreaks (Table 3). Serotype 4b caused the largest number of outbreaks (n = 10) and outbreak-associated cases (n = 218). Serotype 4b was also associated with the highest hospitalization rate (70%) and the highest CFR (13%). Serotype 1/2a was responsible for 8 (40%) outbreaks and 119 (33%) cases. Serotype 1/2b was least common, causing 2 (10%) outbreaks that resulted in 5 (1%) outbreak-associated cases and no deaths (Table 3).

**Table 3:** Characteristics of 24 listeriosis outbreaks by *Listeria monocytogenes* serotype, Foodborne Disease Outbreak Surveillance System, United States, 1998–2008*

Serotype	No. outbreaks	Total no. cases†	Median duration, d (range)	No. cases/outbreak	No. (%) hospitalized	No. deaths (CFR, %)
4b	10	218	68 (1–389)	22	152 (70)	28 (13)
1/2a	8	119	99 (3–351)	15	53 (45)	8 (7)
1/2b	2	5	5 (2–7)	3	3 (60)	0
Unknown	4	17	NA	4	7 (41)	2 (12)

*NA, not available. †Includes laboratory-confirmed and epidemiologically linked cases.

In the early study period (1998–2003), 13 listeriosis outbreaks (4 multistate and 9 single-state) were reported, and in the late study period (2004–2008) 11 (3 multistate and 8 single-state) were reported (Figure 1). Information on outbreak duration was available for 5 (38%) of 13 early study period outbreaks and for all late study period outbreaks. Among outbreaks with information available, those reported in the early study period were generally larger than those in the late study period (median 11 cases [range 2–108 cases] vs. 5 cases [range 2–20 cases]) and longer (122 days vs. 36 days). Among the 11 outbreaks with a food vehicle reported in the early study period, 5 were associated with deli meats and 3 with frankfurters. Novel food vehicles (sprouts and taco/nacho salad) were reported only in the late study period.

## Conclusions

**Figure 3:**
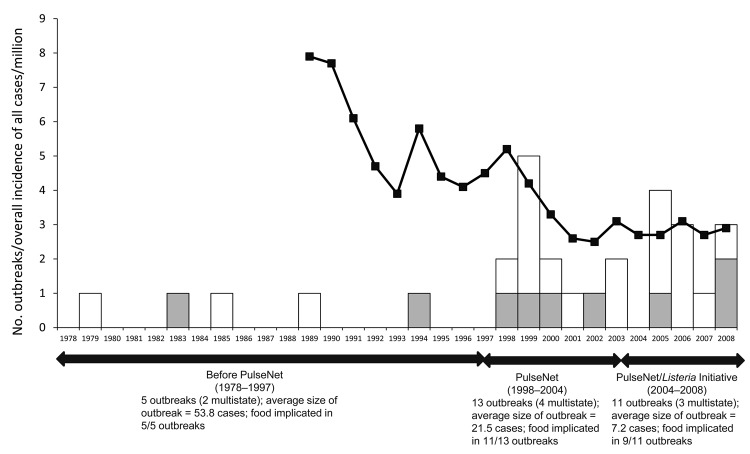
Incidence of all cases (per million) and outbreaks of listeriosis, 1978–2008, United States. White bar sections indicate single-state outbreaks, gray bar sections indicate multisite outbreaks, and black line indicates incidence per million. Data were obtained from the Foodborne Diseases ActiveSurveillance Network (FoodNet) and the Foodborne Disease Outbreak Surveillance System. Data are as of June 2010.

Changes in characteristics of outbreaks reported to CDC during 1998–2008 highlight the successes and continued challenges of listeriosis prevention. During the study period, an average of 2.2 confirmed outbreaks per year were reported. In contrast, an average of 0.25 outbreaks per year was reported during the 20 years before the study period (i.e., the pre-PulseNet era) (Figure 3). We theorize that this 9-fold increase in the number of listeriosis outbreaks reported is predominantly attributed to enhanced detection through PulseNet. Furthermore, outbreaks in the late study period were generally shorter and smaller than those reported in the early study period. This finding suggests that the combined application of PulseNet and the *Listeria* Initiative was useful for timely outbreak detection and investigation (*14*).

Participation in the *Listeria* Initiative, measured by the number of states reporting >1 cases and the percentage of cases reported among all nationally notified cases, has increased steadily from 2004 (no. states reporting = 10, percentage of cases reported = 15%) to 2010 (no. states reporting = 42; percentage of cases reported = 71%) (CDC, unpub. data). The *Listeria* Initiative has contributed to numerous investigations of outbreaks, including those attributed to pasteurized milk, tuna salad served in a hospital, Mexican-style cheese, and hog head cheese (a meat jelly made from swine heads and feet) (*20*,*23*–*25*).

Changes in the size and duration of listeriosis outbreaks occurred during a time when the national incidence of invasive listeriosis decreased by 24% during 1996–2003 (*5*), and then reached a plateau during 2004–2009 (*6*). Enhanced regulatory and industry efforts that stemmed from outbreak investigations (*15*) likely contributed in great part to the observed decrease in invasive listeriosis cases during this earlier time. Notably, 8 of 9 outbreaks associated with frankfurters or deli meat occurred early in the study period. The marked reduction in outbreaks associated with ready-to-eat (RTE) meat and poultry mirrored a decrease in the occurrence of *L.* *monocytogenes* contamination in RTE meat and poultry, from 1.0% to 8.1% in the 1990s to 0.3% in 2010 (*26*).

The reduction in *L. monocytogenes*–contaminated RTE meat and poultry likely demonstrates the effect of several federal regulatory initiatives. First, the US Department of Agriculture Food Safety and Inspection Service (USDA-FSIS) required in 1999 that hazard analysis and critical control point systems be reassessed to ensure that meat and poultry processing establishments in the United States were adequately addressing risk for infection with *L. monocytogenes*. Second, in 2003, USDA-FSIS issued the *Listeria* Rule, which encourages establishments to use combinations of postlethality treatments (i.e., after the product has been cooked), such as inclusion of antimicrobial drugs and bacterial growth inhibitors, to prevent contamination (*27*). Most large producers of frankfurters and fully cooked sliced meat and poultry products, such as deli meat, use >1 such interventions (USDA-FSIS, unpub. data); establishments that opt to forgo them are subject to requirements for more frequent product and production surface sampling (*28*). Third, USDA-FSIS implemented a random sampling program in 2004 for *L. monocytogenes* in production establishments; an additional risk-based, verification sampling program with compliance incentives was started in 2005 (*29*). Establishments where *L.* *monocytogenes* is detected undergo intensive sampling (intensified verification testing) as part of an assessment of the ability of the establishment to manufacture products eligible to bear the mark of inspection (*30*). Although an outbreak of listeriosis associated with deli meat (hog head cheese) occurred in 2010 (*25*), the processing establishment was subject only to state, not federal, regulation because it did not distribute its product outside the state.

In contrast, listeriosis outbreaks from dairy products showed no decrease in frequency throughout the study period. Unpasteurized (raw) milk poses a relatively high risk for listeriosis on a per-serving basis. Mexican-style cheese made from raw milk contributed to at least 1 dairy-associated listeriosis outbreak in the study period (*22*). Although certain cheeses made from raw milk that are aged for >60 days can be sold under Food and Drug Administration regulations (21 CFR Section 133), outbreaks of bacterial, enteric diseases associated with aged cheeses have occurred (*31*), and microbiologic studies have demonstrated the viability of bacterial pathogens in cheeses made from raw milk that have aged >60 days (*32*). In addition to outbreaks associated with cheese made from raw milk, listeriosis outbreaks from pasteurized dairy products (*20*) also occurred during the study period. Although pasteurization is known to adequately eliminate *L.* *monocytogenes* from dairy products, postpasteurization contamination can occur and was the likely source of contamination in at least 1 of the listeriosis outbreaks (*24*) during the study period and at least 4 additional listeriosis outbreaks associated with Mexican-style cheese since the study period (*33*). To prevent postpasteurization contamination, it is necessary to maintain strict separation of prepasteurized and postpasteurized dairy products and strict sanitation in the postprocessing environment.

The outbreaks associated with Mexican-style cheese primarily affected women 1–49 years of age, which includes the reproductive years. FDOSS does not collect information on ethnicity or pregnancy status, but other surveillance data indicate that pregnant Hispanic women have disproportionately higher rates of listeriosis (*6*). Investigations of outbreaks associated with Mexican-style cheese have informed recommendations for pregnant women to avoid consumption of unpasteurized dairy products, including Mexican-style cheeses.

The Food Safety Modernization Act provides the Food and Drug Administration with greater authority to prevent and control outbreaks of listeriosis and other foodborne infections through mandatory prevention controls and safety standards, more stringent tools for inspection and compliance, and an enhanced ability to remove contaminated foods from the food supply (e.g., mandatory recalls). However, dairy products imported from countries with different regulatory controls, homemade products or products made by unlicensed manufacturers, and products contaminated after pasteurization continue to pose regulatory and enforcement challenges (*22*). Therefore, additional prevention and education efforts are still needed to effectively protect susceptible populations.

Several novel food vehicles, including sprouts and taco/nacho salad, were implicated in listeriosis outbreaks described in this review. Three additional novel vehicle outbreaks have since been reported. In 2010, hog head cheese was associated with 8 listeriosis cases in Louisiana (*25*), and celery in a diced chicken salad was associated with an outbreak of 10 cases among hospitalized patients in Texas (*34*). Whole cantaloupe from a single farm was associated with a large, multistate outbreak (n = 147 cases) in 2011, and caused the most deaths (n = 33) from an outbreak of foodborne illness in the United States in nearly 90 years (*16*). Outbreaks that were linked to food vehicles with multiple ingredients are noteworthy because identifying a particular ingredient that is contaminated can be challenging (e.g., celery in chicken salad, taco/nacho salad). Preventing *L. monocytogenes* contamination of food is difficult; the bacteria proliferate under acidic conditions, high salt concentrations, and low temperatures. We expect that future outbreak investigations will continue to identify new food vehicles that can transmit *L. monocytogenes* to humans and identify new opportunities for consumer prevention education, industry interventions to reduce contamination, and regulatory control policies.

We found that serotype 4b caused more outbreaks, more outbreak-associated cases, and resulted in higher rates of hospitalization and deaths than serotypes 1/2a and 1/2b. Studies have shown that most isolates from food belong to serogroup 1/2, but most epidemic listeriosis is caused by serotype 4b (*35*,*36*). In addition, a study in Denmark suggested that illness caused by serogroup 4 is associated with a higher CFR than serogroup 1/2 (*37*). Recent research on *L.* *monocytogenes* virulence mechanisms has shown that certain mutations in the *inlA* gene, which encodes for internalin (a membrane-anchored protein responsible for *L. monocytogenes* invasion into nonprofessional phagocytes), can attenuate virulence (*38*,*39*). These virulence-attenuating mutations are prevalent in serogroup 1/2 but have not been observed in serotype 4b isolates (*40*). Further work is needed to bridge our understanding of *L. monocytogenes* virulence to epidemiologic trends in sporadic and outbreak-associated listeriosis.

There is wide variation in outbreak reporting practices among FDOSS users. For example, New York reported the largest number of listeriosis outbreaks during the study period, but active, population-based surveillance do not suggest that the incidence of invasive listeriosis was higher in New York than in other states (*5*). These findings may reflect variable population sizes and composition and differences in resources for investigating and reporting outbreaks among states, but more information is needed to fully understand these differences. Variation in the quality and completeness of FDOSS reports can limit conclusions drawn from the data. For example, demographic information was missing for nearly 30% of the outbreaks we analyzed. Because FDOSS uses the same reporting form for all pathogens, variables of specific interest for listeriosis, such as underlying medical conditions, ethnicity, age categories for older adults, pregnancy status, and clinical outcomes, are not available unless the reporting agency submits supplemental information. For example, the fact that 2 outbreaks occurred in hospital settings suggests that the medical fragility of the outbreak populations was a major contributor to the outbreaks. Although clinical information is not collected in FDOSS, there is a recognized need to improve food safety in hospitals (e.g., avoiding service of higher risk foods to immunocompromised patients) (*6*,*23*).

Listeriosis outbreak investigations are crucial to prevent additional illness, hospitalization, and death. They also provide a unique source of information to improve our understanding of *L. monocytogenes* infections and transmission and to identify gaps in industry and regulatory measures to safeguard against contamination of the food supply. The changes we observed in characteristics of listeriosis outbreaks during 1998–2008 illustrate the contributions of PulseNet and the *Listeria* Initiative for outbreak detection and investigation and subsequent effects of industry and regulatory efforts to prevent similar contamination from reoccurring. In particular, we found that reports of outbreaks caused by frankfurters and deli meats have become less frequent. Also, outbreaks have generally become smaller in size and shorter in duration.

Nevertheless, listeriosis outbreaks continue to cause considerable illness and death and associated costs. For example, the 2011 outbreak associated with whole cantaloupe (*16*) demonstrated that large outbreaks can still occur and that work remains to identify and prevent new sources of contamination. Repeated occurrences of outbreaks caused by dairy products, especially Mexican-style cheese, also signal the need for additional work. Although invasive listeriosis is rare (average annual incidence for 2004–2009 was 0.27 cases/100,000 population) (*6*), US subpopulations at increased risk are growing in size, including adults ≥65 years of age and Hispanics (2010 Census estimates of 40.3 and 50.5 million, respectively). Therefore, a concerted effort from regulatory, industry, and public health authorities will be required to reduce the overall incidence of listeriosis below the Healthy People 2020 goal of 0.2 cases per 100,000 population (www.healthypeople.gov).
